# Plasma biomarkers for neurodegenerative disorders: ready for prime time?

**DOI:** 10.1097/YCO.0000000000000851

**Published:** 2023-01-16

**Authors:** Wasiu G. Balogun, Henrik Zetterberg, Kaj Blennow, Thomas K. Karikari

**Affiliations:** a Department of Neurosciences, School of Medicine, Case Western Reserve University, Cleveland, Ohio, USA; b Department of Psychiatry and Neurochemistry, Institute of Neuroscience and Physiology, The Sahlgrenska Academy, University of Gothenburg, Gothenburg; c Clinical Neurochemistry Laboratory, Sahlgrenska University Hospital, Mölndal, Sweden; d Department of Neurodegenerative Disease, UCL Institute of Neurology; e UK Dementia Research Institute at UCL, London, UK; f Hong Kong Center for Neurodegenerative Diseases, Hong Kong, China; g Wisconsin Alzheimer's Disease Research Center, University of Wisconsin School of Medicine and Public Health, University of Wisconsin-Madison, Madison, Wisconsin; h Department of Psychiatry, School of Medicine, University of Pittsburgh, Pittsburgh, PA, USA

**Keywords:** Alzheimer's disease, Alzheimer's disease and related disorders, dementia, neurodegenerative disorder, plasma biomarker

## Abstract

Several plasma biomarkers for Alzheimer's disease and related disorders (ADRD) have demonstrated clinical and technical robustness. However, are they ready for clinical implementation? This review critically appraises current evidence for and against the immediate use of plasma biomarkers in clinical care.

**Recent findings:**

Plasma biomarkers have significantly improved our understanding of ADRD time-course, risk factors, diagnosis and prognosis. These advances are accelerating the development and in-human testing of therapeutic candidates, and the selection of individuals with subtle biological evidence of disease who fit the criteria for early therapeutic targeting. However, standardized tests and well validated cut-off values are lacking. Moreover, some assays (e.g., plasma Aβ methods) have poor robustness to withstand inevitable day-to-day technical variations. Additionally, recent reports suggest that common comorbidities of aging (e.g., kidney disease, diabetes, hypertension) can erroneously affect plasma biomarker levels, clinical utility and generalizability. Furthermore, it is unclear if health disparities can explain reported racial/ethnic differences in biomarker levels and functions. Finally, current clinically approved plasma methods are more expensive than CSF assays, questioning their cost effectiveness.

**Summary:**

Plasma biomarkers have biological and clinical capacity to detect ADRD. However, their widespread use requires issues around thresholds, comorbidities and diverse populations to be addressed.

## INTRODUCTION

The development of blood-based biomarkers for Alzheimer's disease (AD) and related neurodegenerative disorders (ADRD) is ground-breaking, as they may help to improve biological understanding of these diseases and to accelerate screening (risk prediction) in clinical management and may also be useful for prognostication [1–4]. Blood biomarkers may also enable evaluation of the efficacy of candidate pharmacological and nonpharmacological agents, assessment of future disease risk in asymptomatic individuals, and longitudinal monitoring of people with symptoms [1–4].

Plasma biomarkers are anticipated to be simpler, more cost-effective, and easier-to-implement alternatives to cerebrospinal fluid (CSF) and neuroimaging biomarkers that are now the most established methods for clinical and research-based assessments of ADRD [1,3–5]. The core CSF biomarkers (β-amyloid [Aβ]_42_/Aβ_40_ ratio, phosphorylated tau 181 [p-tau], and total tau) jointly perform excellently to provide biological evidence of AD, in agreement with the principal pathological features of the disease – Aβ plaques, phosphorylated tau, and neurodegeneration respectively [5–7]. Neuroimaging alternatives to these CSF biomarkers respectively include positron emission tomography (PET) imaging of Aβ plaques and tau tangles, as well as magnetic resonance imaging (MRI) of hippocampal atrophy [5–7]. These markers are included in diagnostic and research guidelines and some are approved for clinical use by the US Food and Drugs Administration [5–8]. Yet, their invasiveness, high costs and difficulty to upscale hinder their widespread applications in primary care [1,2].

Technical developments have led to the development of plasma biomarkers that require minimal expertise in sample collection. Plasma biomarkers have additional potential advantages including suitability for prescreening and lower costs that are important for large-scale clinical diagnostic, prognostic, interventional and observational applications [1–4,9^▪▪^].

We provide a short up-to-date review on recent findings in favor of plasma biomarkers as the next generation of accurate diagnostic and prognostic biomarkers to detect AD neuropathologic change. We additionally discuss the counterargument that despite their demonstrably high performances, widespread clinical use would require that specific issues that are critical to these endeavors are first and foremost addressed.

## HIGH-PERFORMING BLOOD BIOMARKERS FOR NEURODEGENERATIVE DISORDERS: AN UPDATE

Several blood biomarkers have shown utility for AD and ADRD. In this review, we focus on biomarkers that have shown repeated utility across multiple independent studies. Plasma neurofilament light chain (NfL), an indicator of axonal injury/neurodegeneration, is probably the most widely used blood biomarker. Being a universal biomarker of a disease feature common to multiple neurodegenerative pathologies, plasma NfL levels are higher not only in AD but also in several other neurodegenerative diseases including amylotrophic lateral sclerosis, frontotemporal lobal degeneration and primary tauopathies such as progressive supranuclear palsy and corticobasal syndrome [10^▪▪^,^11^]. Other biomarkers include plasma Aβ42/Aβ40 and p-tau that have shown potential for AD detection. Plasma Aβ42/Aβ40 associates with brain Aβ pathology [12,13^▪▪^,^14^], whereas p-tau biomarkers (including p-tau181, p-tau217 and p-tau231) are known to increase according to Aβ and tau pathophysiologies [9^▪▪^,^15^,^16^^▪▪^,^17^^▪▪^]. In addition, plasma p-tau may predict future cognitive impairment [9^▪▪^,^18^^▪▪^,^19^^▪▪^,^20^^▪▪^]. It is highly concordant with AD diagnosis at autopsy [21,22^▪▪^]. The presence of astroglia activation in AD [23^▪▪^,^24^^▪▪^,^25^^▪▪^], as well as in non-AD disorders, such as multiple sclerosis and frontotemporal lobar degeneration, support the use of plasma glial fibrillary acidic protein (GFAP) as a blood biomarker [26,27^▪^]. 

**Box 1:**
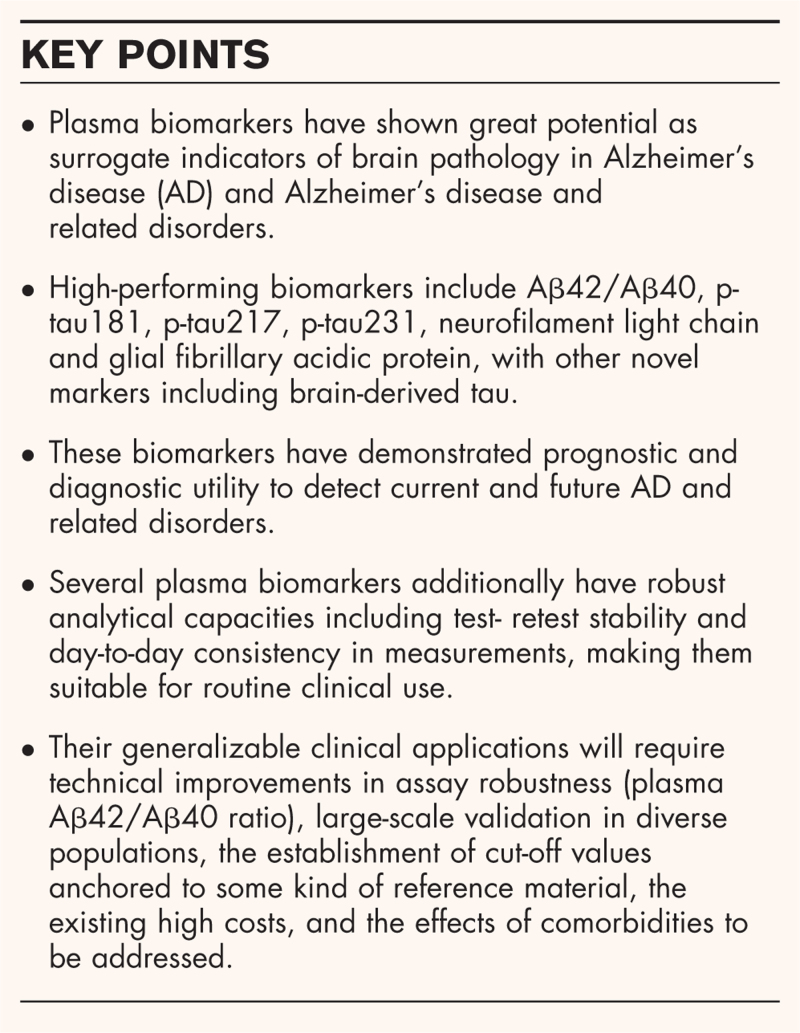
no caption available

## DIAGNOSTIC AND PROGNOSTIC PERFORMANCES SUGGESTING THAT BLOOD BIOMARKERS ARE READY FOR PRIME TIME

NfL: In most neurodegenerative disorders, plasma NfL levels are elevated because axonal degeneration is a prominent feature [28,29]. Individuals with AD across the disease spectrum – asymptomatic AD, prodromal AD, and familial AD – show elevated blood levels of NfL [10^▪▪^,^30^–^32^]. The concentration of NfL in blood inversely associates with cognitive function, and positively with CSF biomarkers, postmortem pathology, and imaging findings [10^▪▪^]. Furthermore, plasma NfL predicts longitudinal disease outcome including progression from asymptomatic to symptomatic phase [33–36]. Plasma NfL associates with MRI signatures of neurodegeneration [34,37], as well as the severity of neurodegeneration at autopsy [29]. Additionally, NfL is analytically robust, with highly reproducible day-to-day values when measured in either plasma or serum [38^▪^,39^▪^].

Aβ42/Aβ40: One head-to-head comparison study suggested that plasma Aβ42/Aβ40 measured by immunoprecipitation-mass spectrometry (IP-MS) technology was the best-performing to detect brain Aβ compared with immuno-assay methods [40^▪▪^], although immunoassay methods including the ones from Quanterix and Roche recently showed considerably good performance for early detection of Aβ pathology [20^▪▪^,^41^^▪▪^]. Plasma Aβ42/Aβ40 associates well with, and predicts longitudinal changes in brain Aβ PET and CSF Aβ42/Aβ40 [12,13^▪▪^,^14^,42^▪^]. Moreover, plasma Aβ42/Aβ40 is one of the biomarkers that starts to change in the early preclinical phase of AD [12,13^▪▪^,^14^]. This biomarker also has only a small fold change between Aβ-positive and Aβ-negative individuals when compared with CSF Aβ42/Aβ40 [1,12], making the biomarker not robust in everyday laboratory practice [43^▪▪^]. Plasma Aβ42/Aβ40 performs well when samples are analyzed batch-wise with single lots of assay reagents.

P-tau: Several plasma p-tau species have been developed and validated, with p-tau181, p-tau217 and p-tau231 being the most well studied [1,4,44]. There is strong evidence that plasma p-tau is a reliable biomarker for AD, with demonstrated utility in multiple clinical contexts; these include AD time course [45], definitive diagnosis and differential diagnosis versus other causes of cognitive impairment [15,16^▪▪^,^17^^▪▪^,^21^,^22^^▪▪^,46^▪^], disease prognosis in primary care [15,17^▪▪^], and in participant selection and efficacy monitoring in therapeutic trials [18^▪▪^,^20^^▪▪^]. These performances have been independently authenticated in dozens of research cohorts, with some studies further showing that plasma p-tau measures correlate well with CSF p-tau in paired samples [1,4,44]. Moreover, plasma p-tau often performs equivalently to CSF p-tau to differentiate Aβ-positive AD dementia individuals from Aβ-negative non-AD dementia and control participants [16^▪▪^,^47^^▪▪^]. The specificity of plasma p-tau to AD pathophysiology (compared with other biomarkers like NfL and GFAP) makes it a potential first line of action in the diagnostic workup [1,2]. Moreover, plasma p- tau shows larger fold change between symptomatic AD patients and controls compared with plasma Aβ42/Aβ40, which leads to high test-retest reproducibility and robustness, supporting utility in clinical laboratory practice [38^▪^,^48^^▪▪^].

GFAP: GFAP is an intermediate filament protein highly expressed in astrocytes whose main physiological function is to provide network support and structure to cells [49]. Plasma GFAP associates with *in vivo* Aβ pathology across the AD continuum [23^▪▪^,^24^^▪▪^,^50^]. Plasma GFAP associates better with Aβ-PET than CSF GFAP [24^▪▪^]. The higher preanalytical stability of plasma versus CSF GFAP partly but not fully explains this observation [51^▪^], with the further speculation that plasma GFAP levels may be affected by blood-brain barrier dysfunction. Beyond AD, a rise in GFAP levels in frontotemporal dementia may indicate the late presymptomatic stage, as well as the severity of the disease [26,27^▪^]. Plasma GFAP is also increased in neuroinflammatory conditions, including multiple sclerosis, and is a top biomarker candidate for the progressive form of the disease [52,53].

Novel plasma total-tau (t-tau) biomarkers: Recently reported plasma biomarkers of clinical value include those that have sought to develop improved t-tau assays in blood. Similar to plasma p-tau methods that target N-terminal tau protein fragments that seem to be more abundant in blood compared with mid-region and C-terminal forms [1,54], the development of t-tau assays have capitalized on the same approach. The NT1 assay [55] targets tau molecular forms containing amino acids (aa) 6–198 by pairing the antibodies Tau12 (epitope: 6–18) with BT2 (aa 194–198).

Another assay, tau NTA, targets an even shorter N-terminal-bearing fragment, and tends to be increased earlier in the disease process [56^▪▪^]. Both of these outperform the existing plasma t-tau assay from Quanterix [55,56^▪▪^]. More recently, a new plasma t-tau assay that is specific to tau of brain-origin was described [57^▪▪^]. This assay – referred to as brain-derived tau (BD-tau) – avoids tau of peripheral origin, with levels in plasma and CSF correlating strongly; a strong correlation is also seen between plasma BD-tau and CSF t-tau [57^▪▪^]. Plasma BD-tau associates well with Aβ and tau pathology *in vivo* and at autopsy, and also differentiates AD from non-AD neurodegenerative diseases [57^▪▪^].

## FINDINGS SUGGESTING THAT BLOOD BIOMARKERS ARE NOT READY FOR PRIME TIME

Blood biomarkers represent a paradigm shift and game changer in the AD field. However, there are still some issues to address to enable their widespread use and acceptance. These include the following points:

### Analytical sensitivity

Despite their proven capacity to measure pico- to femto-molar quantities of brain proteinopathies in remote blood, some of the existing biomarker methods have limitations for the accurate detection of very low levels of their target analytes [1]. Since AD develops slowly over several years to a decade or possibly beyond, disease prevention and treatment strategies will benefit greatly from plasma biomarkers that can accurately identify disease risk several decades before older adulthood. One of such markers with sensitivity limitation is p-tau217 which is otherwise highly effective at detecting AD pathology [1].

### Lack of assay standardization

Certified reference methods and materials for assay standardization are lacking for all of the biomarkers reviewed in this paper. This puts high demands on assay manufacturers to produce kits with low lot-to-lot variation. Laboratories using these assays must also implement control programs through which longitudinal stability of the measurements in relation to the studies in which reference limits and cut-offs were established is monitored and maintained.

### Lack of validated abnormality thresholds

Generalized, multicenter application will require that plasma biomarkers have been vigorously validated to generate cut-off points (traceable to some type of reference material) that work well across populations, similar to what is currently available for neuroimaging and CSF biomarkers.

### Technical robustness

Plasma p-tau, NfL, and GFAP have wide analytical ranges, large fold changes between diagnostic groups, are not significantly affected by preanalytical handling factors, and thus demonstrate strong technical robustness that can withstand small day-to-day measurement biases [38^▪^,39^▪^,^48^^▪▪^,^58^]. However, plasma Aβ42/Aβ40 – whether measured by IP-MS or by immunoassay methods – has small fold changes and narrow analytical range that are susceptible to preanalytical variations [1,38^▪^,^43^^▪▪^,^48^^▪▪^].

### Cost

Recent simulation analyses estimated the cost of a single plasma biomarker testing to be as low as $50 to drive significant cost-savings compared with CSF and neuroimaging [9^▪▪^,^13^^▪▪^]. However, the cost of approved tests or diagnostic use in the United States is currently much higher than this value. An example is the PrecivityAD test from C2N Diagnostics, which combines plasma Aβ42/Aβ40 ratio with age and *APOE* ε4 genotype information to predict brain Aβ load. This test costs $1250 per analysis, which is almost half the average cost of Aβ PET imaging [59]. Another plasma Aβ42/Aβ40 ratio test from Quest Diagnostics, which is based on a CSF assay [60] with not much having been published on the plasma version, is believed to cost about $500. Importantly, both methods are more expensive than the FDA-approved CSF Aβ42/Aβ40 ratio test available from Lumipulse, questioning the cost advantage argument often put forward in favor of plasma biomarkers.

Research cohort composition not reflecting the wider population: The demographics of participants in research cohorts among whom biomarker testing is performed are uneven, with the majority of cohort studies in the United States focusing on middle-class non-Hispanic whites. To this end, individuals of other demographics – including self-identified racial/ethnic groups, other socioeconomic statuses, and those living in disadvantaged areas – need to be actively included to ensure that the results obtained are generalizable to the larger population [61].

### Differences in biomarker levels and performances between populations

A few reports have suggested that plasma biomarker levels and performances tend to differ between participants of different ethnoracial backgrounds [62^▪▪^,^63^^▪▪^] whereas another study did not report any differences [64^▪▪^]. Importantly, other studies have pointed to a likelihood that the intensity of brain pathological changes appear to be less pronounced in participants of non-European ancestry who also tend to be less affected by the presence of the major genetic risk *APOE* ε4 [65^▪▪^,^66^]. These results need to be actively investigated to, among other things, identify potential disease resilience/resistance factors.

### Effects of comorbidities

A diagnosis of common comorbidities of aging – particularly those that affect organs where tau protein is highly expressed (e.g., kidney disease, hypertension, diabetes) – can erroneously affect plasma biomarker levels and clinical performances [67^▪▪^,^68^^▪▪^]. On the other hand, autopsy-verified mixed dementias are more common in Black populations versus non-Hispanic White individuals [69]. However, it is unknown if and how these multimorbidities affect biomarker accuracies. It is imperative to comprehensively evaluate effects of these and a wider spectrum of comorbidities in diverse populations round the world [61].

## CONCLUSION

Much effort has been made to develop and clinically validate several plasma biomarkers, including plasma Aβ42/Aβ40, p-tau181, p-tau217, p-tau231, NfL, and GFAP. These markers have shown immense diagnostic and prognostic utility to detect AD and ADRD in multiple independent cohorts. Given their ability to identify pathophysiological disease changes including when compared with autopsy diagnosis, and that most have high preanalytical stability, these markers are appropriate for clinical and prognostic applications. Nonetheless, issues such as assay standardization, the establishment of cut-off values, technical robustness (particularly for Aβ42/Aβ40 ratio), high costs, large-scale validation in diverse populations, and the effects of comorbidities need to be addressed to enable fuller understanding and generalizability of findings.

## Acknowledgements

*We thank all researchers, study participants, their families and friends, funders, patient organizations, and pharma and biotech companies that have taken part in generating the data that was reviewed here.*

### Financial support and sponsorship

*T.K.K. was funded by the National Institute on Aging (R01 AG053952-05), Swedish Research Council (Vetenskapsrådet #2021-03244), the Alzheimer's Association Research Fellowship (#AARF-21-850325), the Aina (Ann) Wallströms and Mary-Ann Sjöbloms stiftelsen, and the Emil och Wera Cornells stiftelsen. HZ is a Wallenberg Scholar supported by grants from the Swedish Research Council (#2018–02532), the European Union's Horizon Europe research and innovation programme under grant agreement no. 101053962, Swedish State Support for Clinical Research (#ALFGBG-71320), the Alzheimer Drug Discovery Foundation (ADDF), USA (#201809-2016862), the AD Strategic Fund and the Alzheimer's Association (#ADSF-21-831376-C, #ADSF-21-831381-C, and #ADSF-21-831377-C), the Bluefield Project, the Olav Thon Foundation, the Erling-Persson Family Foundation, Stiftelsen för Gamla Tjänarinnor, Hjärnfonden, Sweden (#FO2022-0270), the European Union's Horizon 2020 research and innovation programme under the Marie Skłodowska-Curie grant agreement no. 860197 (MIRIADE), the European Union Joint Programme – Neurodegenerative Disease Research (JPND2021-00694), and the UK Dementia Research Institute at UCL (UKDRI-1003). K.B. is supported by the Swedish Research Council (#2017-00915 and #2022-00732), the Swedish Alzheimer Foundation (#AF-930351, #AF-939721 and #AF- 968270), Hjärnfonden, Sweden (#FO2017-0243 and #ALZ2022-0006), the Swedish state under the agreement between the Swedish government and the County Councils, the ALF-agreement (#ALFGBG-715986 and #ALFGBG-965240), the European Union Joint Program for Neurodegenerative Disorders (JPND2019-466-236), the Alzheimer's Association 2021 Zenith Award (ZEN-21-848495), and the Alzheimer's Association 2022−2025 Grant (SG-23–1038904 QC).*

### Conflicts of interest

*W.G.B. and T.K.K. have no conflicts of interest. HZ has served at scientific, advisory boards and/or as a consultant for Abbvie, Acumen, Alector, Alzinova, ALZPath, Annexon, Apellis, Artery Therapeutics, AZTherapies, CogRx, Denali, Eisai, Nervgen, Novo Nordisk, Optoceutics, Passage Bio, Pinteon Therapeutics, Prothena, Red Abbey Labs, reMYND, Roche, Samumed, Siemens Healthineers, Triplet Therapeutics, and Wave, has given lectures in symposia sponsored by Cellectricon, Fujirebio, Alzecure, Biogen, and Roche, and is a co-founder of Brain Biomarker Solutions in Gothenburg AB (BBS), which is a part of the GU Ventures Incubator Program (outside submitted work). K.B. has served as a consultant, at advisory boards, or at data monitoring committees for Abcam, Axon, BioArctic, Biogen, JOMDD/Shimadzu. Julius Clinical, Lilly, MagQu, Novartis, Ono Pharma, Pharmatrophix, Prothena, Roche Diagnostics, and Siemens Healthineers, and is a co-founder of Brain Biomarker Solutions in Gothenburg AB (BBS), which is a part of the GU Ventures Incubator Program, outside the work presented in this paper.*

## REFERENCES AND RECOMMENDED READING

Papers of particular interest, published within the annual period of review, have been highlighted as:

▪ of special interest
▪▪ of outstanding interest
